# Effects of Vitamin D supplementation on resolution of fever and cough in children with pulmonary tuberculosis: A randomized double-blind controlled trial in Indonesia

**DOI:** 10.7189/jogh.12.04015

**Published:** 2022-02-18

**Authors:** Lianda Tamara, Cissy B Kartasasmita, Anggraini Alam, Dida A Gurnida

**Affiliations:** 1 Department of Child Health, Faculty of Medicine, Universitas Padjadjaran, Bandung, West Java, Indonesia; 2 Bethesda Serukam Hospital, Bengkayang, West Borneo, Indonesia

## Abstract

**Background:**

Various studies in adults have shown a strong association between vitamin D and tuberculosis (TB), both in terms of vitamin D status and the benefits of vitamin D in managing TB. Studies on vitamin D and its relationship with childhood TB still lack in Indonesia as a country with the second-highest TB incidence globally. This study evaluated the effect of vitamin D supplementation on resolution of cough and fever in Indonesian children with pulmonary TB.

**Methods:**

We conducted a randomized controlled trial of vitamin D supplementation in children with pulmonary TB and vitamin D insufficiency. Patients were randomly allocated with 1:1 ratio to receive either 1000 IU vitamin D or placebo daily after starting standard TB treatment. The primary outcome in this study was the resolution of fever and cough symptoms reviewed weekly after starting the treatment until the symptoms are resolved. The secondary outcome in this study was 25-hydroxyvitamin D serum level and nutritional status which was reviewed at the end of the trial. Intention to treat analyses were applied. Differences in clinical outcomes between two groups were calculated using Mann-Whitney U test or χ^2^ test, where appropriate.

**Findings:**

A total of 84 patients met the inclusion criteria, aged 6 to 18 years old, newly diagnosed with pulmonary TB and vitamin D insufficiency. Eighty patients (95,2%) completed the six months follow-up. Faster resolution of fever, cough, improved malnutrition status, and higher vitamin D level were found in the intervention group compared to the placebo group (all *P* < 0.001).

**Conclusions:**

Vitamin D is beneficial in improving fever and cough resolution, and improving nutritional status in children with pulmonary TB and vitamin D insufficiency. Determination of adequate supplementation levels of more than 1000 IU requires further research to achieve normal vitamin D levels during the duration of treatment for pulmonary TB in children.

**Trial registration:**

ClinicalTrials.gov (NCT05073965).

Globally in 2019, an estimated 10.0 million people were diagnosed with TB in which most of the TB cases were in the World Health Organization (WHO) regions of South-East Asia (44%) [1]. Indonesia accounted for 845.000 TB cases, ranked as the second-highest TB incidence in the world [1]. Despite the inconsistent quality of notification data, children (aged <15 years) accounted for 12% (1.2 million) of the global TB cases [1]. The focus and development of methods for estimating the global burden of childhood TB has overgrown in recent years, followed by various studies supporting the management of childhood TB [2].

Some systematic reviews on vitamin D and TB found positive and negative results. The negative findings revealed no correlation between vitamin D levels and TB and no effect on improving some TB clinical outcomes [3,4]. The positive findings support vitamin D supplementation for TB patients, vitamin D as a predictor of TB disease risk, and a link between vitamin D level and TB [5-8]. However, no definitive conclusion was drawn regarding vitamin D's role in TB, and more research was required. Various studies in have shown association between vitamin D and TB, both in terms of vitamin D status and the benefits of vitamin D in managing TB [6,9-15]. Most of those studies were conducted in the adult population, although several studies have been followed in children [16-20]. A limited study explicitly examines vitamin D and its association with TB in Indonesian children. One of them is a study related to vitamin D status in under-five children with a history of close TB contact, but the study did not address vitamin D status in confirmed TB patient [21]. Based on the geographically dominant distribution of TB incidence in Indonesia, research conducted in Indonesia will significantly contribute to patient care and scientific considerations for policy makers in TB elimination.

Indonesia has a high prevalence of TB and areas with scarce resources that leads to difficulty evaluating the diagnosis and outcome of TB treatment in children. The Indonesian Pediatric Society has developed a scoring system for pediatric TB diagnosis and treatment evaluation in children that incorporates symptoms of cough, fever, and nutritional status as assessment points for clinical TB in children. These parameters can indicate TB therapeutic response in children, particularly among those diagnosed without bacteriological confirmation in limited health care resource. Evaluating the effect of vitamin D supplementation in low resource setting is also complicated. Therefore, using the symptoms improvement, ie, cough and fever, in low resource but high burden TB setting will be significant and practical to evaluate the effect of vitamin D supplementation in children with TB. This study evaluated the effect of vitamin D supplementation on resolution of fever and cough in Indonesian children with pulmonary TB.

## METHODS

This randomized, double-blind control trial was conducted in Bethesda Serukam Hospital West Borneo from December 2020 to May 2021. This trial was registered in ClinicalTrials.gov (NCT05073965).

Study participants were recruited from patients who came to Bethesda Serukam Hospital as suspected TB referrals from the community health center. Patients 6 to 18 years old were assessed for pulmonary TB and vitamin D insufficiency or deficiency as the eligibility to participate. Diagnosis of TB case was based on The Indonesian Pediatric Tuberculosis Scoring System [22]. The scoring system was based on history taking, common TB clinical findings such as fever and cough, laboratory (tuberculin skin test, Gram staining from sputum or gastric lavage), and radiology (chest x-ray) results. Scoring ranges from 0 to 3 for each variable; a score of more than six from a fourteen maximum score is considered a TB diagnosis [22]. The isolation of Mycobacterium tuberculosis (MTB) was not done in this study because of limited resources. Subjects were excluded if they were known of having liver or kidney abnormalities, immunocompromised, or already received vitamin D supplementation before the study. Subject selection was determined based on consecutive sampling according to which patients came to the clinic until the minimum number of samples (84 subjects) met the inclusion criteria. An explanation of the study's aims, procedures, and risks was given orally and in writing to parents and children before they gave written consent.

After obtaining written consent from the parents or guardians, all subjects had their blood specimens drawn to measure serum active 25-hydroxyvitamin D using the enzyme-linked immunosorbent assay (ELISA) method. Vitamin D is categorized as deficiency if serum 25-hydroxyvitamin D is below 20ng/mL, insufficiency between 20─30ng/mL, and normal levels above 30ng/mL [16]. For each subject, the following data were entered into the study database: identification code, age, sex, physical examination results, cough symptom, fever symptom, and body weight. Data about the tests performed were also recorded included vitamin D serum levels (25-hydroxyvitamin D), tuberkulin skin test (TST), chest X-Ray (CXR), gastric or sputum aspiration (GeneXpert for MTB), and anti-tuberculosis treatment. All results were recorded in a study database following international standards to protect the privacy and personal information.

Subjects were randomly assigned to receive either a 1000 IU vitamin D supplement dose or a placebo with an allocation ratio of one to one. Before starting recruitment, the project manager prepared 84 packs of study preparation - 42 packs of the active study drug and 42 packs of a placebo, then generated a randomization sequence using a computer program assigning the terms active or placebo to numbers 1 to 84. The packs were then assigned a randomized number according to this computer-generated randomization sequence.

At recruitment, study staff enrolled patients consecutively according to the order of arrival of patients from number 1 to 84. Study staff who assigned patients to active drugs or placebo did not know the following assignment in the sequence because they did not have access to the study code. Treatment of vitamin D or placebo allocations were hidden from patients and research staff. All subjects received antimicrobial treatment for TB drugs in the form of a fixed-dose combination.

Monitoring of the subject's medication adherence and daily symptoms (cough and fever) was conducted using a checklist table filled out by the patient's parents and confirmed by the researcher when the patient has a follow-up schedule to the clinic. Patients were reviewed for cough and fever symptoms weekly after starting antimicrobial treatment until the symptoms resolved. At each time point, standardized diagnostic measurements such as overnight sputum specimens were collected from all patients who could make spontaneous expectations, body weight and height were also measured. If the patient could not expel sputum spontaneously, a sputum induction procedure was performed in the hospital. Those standard evaluations were carried out to ensure the safety of subjects during intervention and treatment. The 25-hydroxyvitamin D serum was evaluated at the end of the study (6 months after receiving antimicrobial treatment).

The primary outcome measures in this study are resolution of fever and cough. The fever and cough symptoms were reviewed weekly after starting antimicrobial treatment until the symptoms were resolved, while the anthropometric measurement was reviewed six months after receiving antimicrobial treatment. The secondary outcome measures in this study are 25-hydroxyvitamin D serum level and nutritional status (based on the measurement of change in body weight, change in height, and change in Body Mass Index recorded monthly) reviewed six months after receiving antimicrobial treatment. The study was approved by Universitas Padjadjaran Research Ethics Committee with ethical approval number 1155/UN6.KEP/EC/2020.

A sample of 42 individuals in each group (total of 84 study participants) was needed to provide 90% power to detect difference of time of TB symptoms resolution, according to a preliminary study in West Java, Indonesia. Descriptive statistics were presented as counts and proportions for categorical variables. Means and standard deviations or median and ranges were presented for continuous variables, depending on the data distribution.

To compare baseline characteristics between intervention and control groups, χ^2^ test was applied for categorical variables, while Mann-Whitney U test was applied for continuous variables as the data was not normally distributed. To compare differences in symptoms resolution, Mann-Whitney test was performed. P value less than 0.05 was considered as statistically significance. The analyses were conducted based on intention to treat principles. A last observation carried forward (LOCF) method was conducted on four subjects who did not complete the protocol to complete the data for intention to treat analysis. To minimize bias, the intention to treat analysis results were compared to the per-protocol analysis. Stata 16 (StataCorp LCC, USA) was used for the statistical analyses.

## RESULTS

**Figure 1:**
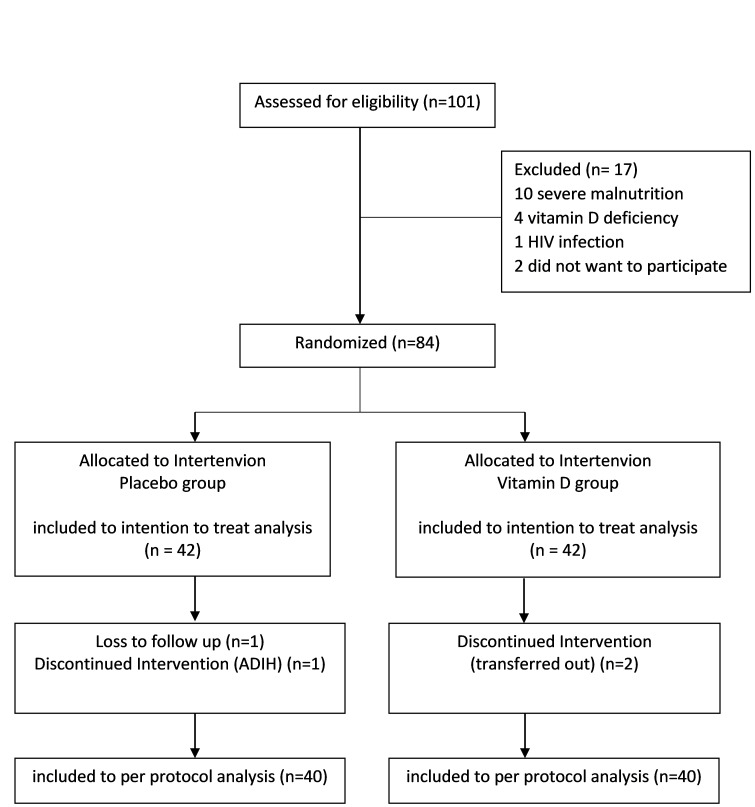
Flowchart of the exclusion process. ADIH – antituberculosis drug-induced hepatotoxicity, HIV – human immunodeficiency virus

We assessed 101 patients for eligibility to participate in the trial: 17 were ineligible, two were eligible but declined, 84 were randomized. Two subjects from the placebo group did not complete the trial due to drug induced hepatotoxicity and loss to follow-up, independently. Two subjects from the intervention group did not finish the trial for continuing treatment in other hospital. Each of the groups had 40 subjects completed the trial. The flowchart is shown in **Figure 1****.**

**Table 1****.** shows no differences in the distribution of sex, age, TB score, and nutritional status before the intervention in the two groups.

**Table 1:** Trial baseline characteristics (n = 84)*

Baseline characteristic	Placebo (n = 42)	Intervention (n = 42)	*P-*value
Sex, n (%)			1.000
Male	24 (57.1)	24 (57.1)	
Female	18 (42.9)	18 (42.9)	
Age (years), median (min-max)	11.5 (6.2-15.6)	9.1 (6-16.8)	0.851
TB Score, median (min-max)	8 (7-10)	8 (7-9)	0.669
Height-for-age z score, median (min-max)	-2.00 (-3.39-0.31)	-1.67 (-3.24-0.08)	0.724
Weight-for-age z score, median (min-max)	-1.67 (-3.3-0.54)	-1.72 (-3.24-0.08)	0.059
BMI-for-age, median (min-max)	-0.70 (-3.32-1.94)	-1.17 (-2.79-0.56)	0.074
Nutritional status, n(%)			0.655
Moderate malnutrition	25 (59.5)	27 (64.3)	
Well nourished	17 (40.5)	15 (35.7)	
Stunting, n (%)			0.103
Severely stunted	2 (4.8)	2 (4.8)	
Stunted	27 (64.3)	19 (45.2)	
Normal	13 (31)	21 (50)	

TB – tuberculosis, BMI – body mass index

*χ^2^ or Mann-Whitney test were applied, where appropriate.

Patient TB work-up and treatment profile are shown in **Table 2****.** Most of the CXR in the subjects of this study showed perihilar lymph node enlargement, both in the placebo group (97.62%) and the intervention group (92.86%). Four study subjects showed positive results of sputum smear examination, two subjects from each group. The use of antituberculosis treatment package in this study was based on Indonesian Pediatric TB management technical guidelines, which is slightly different from WHO fixed dosed combination recommendation regarding the number of tablets per weight band. The antituberculosis treatment package for children category was given to 26 subjects in the placebo group and 32 subjects in the intervention group. The antituberculosis treatment package for adult category was given to 16 subjects in the placebo group and 10 subjects in the intervention group.

**Table 2:** Patient tuberculosis work up and treatment profile (n = 84)

Characteristic	Placebo (n = 42)	Intervention (n = 42)
Chest x-ray:		
Perihilar lymphadenopathy	41	39
Unilateral pleural effusion	1	3
Acid-fast bacilli sputum smear:		
Negative	40	40
Positive	2	2
Molecular test:		
Undetected MTB	40	40
MTB/RIF	2	2
Tuberculin skin test:		
10mm	0	0
≥10mm	42	42
Antituberculosis treatment package category:		
Children	26	32
Adult	16	10

MTB – mycobacterium tuberculosis

**Table 3****.** shows clinical outcomes that fever resolution occurred faster in the intervention group than in the placebo group (*P* < 0.001). The median for fever resolution in the intervention group is 2 weeks compared to 3 weeks in the placebo group. Resolution of cough symptoms also faster in the intervention group than in the placebo group (*P* < 0.001). The median for cough symptoms in the intervention group is 2 weeks compared to 4 weeks in the placebo group.

**Table 3:** Clinical outcomes (n = 84)*

	Placebo (n = 42)	Vitamin D (n = 42)	*P* value*
Fever duration (week)	3 (1-6)	2 (1-3)	<0.001
Cough duration (week)	4 (2-6)	2 (1-6)	<0.001
Nutritional status:			
-Delta BMI-for-age z score	0.08 (-0.47-0.68)	0.5 (-0.62-1.7)	<0.001
-Delta weight-for-age z score	0.33 (0.06-0.9)	0.79 (0.15-1.8)	<0.001
-Delta height-for-age z score	0.55 (0.08-1.02)	0.7 (0.13-1.22)	0.001
Vitamin D levels (ng/mL):			
Before intervention			0.147
Median	22.1	21.7	
Range	20.1-29	20-28.3	
After intervention			0.001
Median	11.2	13.4	
Range	5.7-22.1	10.2-21.5	
Delta vitamin D levels (ng/mL)	-11.35 (-19.5-0)	-8.85 (-15.6-0)	<0.001

BMI – body mass index

* *P*-values were calculated using Mann-Whitney U test.

Nutritional status with delta Body Mass Index (BMI)-for-age z score measurements in the placebo group resulted in a median of 0.08 with a range of -0.47 to 0.68, while in the intervention group, the median was 0.5 with a range of -0.62 to 1.7. Nutritional status with delta weight-for-age z score measurements in the placebo group resulted in a median of 0.33 with a range of 0.06 to 0.9, while in the intervention group, the median was 0.79 with a range of 0.15 to 1.8. Nutritional status with delta height-for-age z score measurements in the placebo group resulted in a median of 0.55 with a range of 0.08 to 1.02, while in the intervention group, the median was 0.7 with a range of 0.13 to 1.22. These results indicate a significant improvement in nutritional status in the intervention group compared to placebo (*P* = 0.001).

Before the intervention, Vitamin D levels in the placebo and intervention groups were similar, ranging from 20 to 29 ng/mL (*P* = 0.147). After receiving the intervention for six months, vitamin D levels in the intervention group was statistically higher than the placebo group (*P* < 0.001). None of the study subjects achieved normal vitamin D levels in either group. After completing the protocol, vitamin D levels in both groups decreased, but the reduction in vitamin D level in the intervention group was statistically lower (*P* < 0.001).

There was a statistically significant difference between the improvement in clinical outcomes in the intervention group compared to the placebo group, based on the duration of fever, duration of cough, and improvement in anthropometric status. The intervention group had a better outcome than the placebo group. No safety issues were found.

## DISCUSSION

This research is the first study in Indonesia to evaluate the effects of vitamin D supplementation on clinical outcomes in children with pulmonary TB. This study highlights that vitamin D improves fever and cough resolutions. Vitamin D also significantly improves the anthropometric status after the end of TB treatment. No safety issues were found.

In this trial, most of the children were symptomatic, while their sputum smear and molecular test were dominantly unconfirmed. These results are in accordance with the previous study that microbiological confirmation of MTB infection is rarely achievable in pediatric pulmonary TB due to difficulty collecting sputum specimens [23]. Most children do not have the expectoration strength and oromotor coordination to produce good quality sputum specimens. All subjects in this study underwent sputum induction, but only four subjects with positive smear results and molecular rapid test results showed sensitivity to rifampin. As microbial examination in children is challenging, monitoring the evolution of the symptoms play important role in TB treatment and aligns with some previous TB studies in children [24].

Fever is a physiological response to various conditions, including TB infection. Based on the 10 years cross-sectional study in Iran, the median duration of the febrile response in adults receiving TB treatment was 11.7 days with a standard deviation of 7.5 [25]. Studies investigating duration of fever in children with TB are still lacking. However, persistent fever was exclusively reported in children with TB, although it was only present in 25% of cases [26]. There is also no research on the relationship between vitamin D and fever as a typical immune response. This study showed that the intervention group experienced significant resolution of fever with a median of 2 weeks compared to the placebo group who experienced a median resolution of fever at 3 weeks. The exact mechanism associated with this is not known. The resolution of the fever may be due to the influence of vitamin D on the immune system in the treatment of TB without excluding the possibility of other variables involved [11,27].

Coughing is also a typical physiological response to a variety of respiratory conditions, including TB infection. A randomized trial in children has shown that most of the children (58%) in the clinical trial setting took longer than 60 days after starting TB treatment for baseline cough to resolve [24]. In this study, the maximum duration of cough was 6 weeks in both groups. The intervention group experienced significant resolution of cough with a median of 2 weeks compared to the placebo group who experienced a median resolution of fever at 4 weeks. A systematic review and meta-analysis of aggregate data from randomized controlled trials showed vitamin D supplementation was safe and slightly reduced the risk of acute respiratory infection [28].

A faster resolution of fever and cough indicates an excellent clinical improvement response. This, of course, affects the quality of life of children and families as the impact of chronic diseases such as TB on children affects physical, psychological, and social health [18]. Faster resolution of fever and cough plays a role in improving the quality of life for children to carry out their daily activities, attend school and interact with friends [29].

Indonesia is one of the countries experiencing the triple burden of malnutrition (undernutrition, micronutrient deficiencies, and overweight) in Asia [30]. Most of the research subjects (62%) were undernourished, in line with the problem of malnutrition and micronutrient deficiencies in general so that the results of this study can be generalized to the Indonesian child population. Studies show proteins and micronutrients play an essential role in developing innate and cellular immunity, and these factors are essential for the TB response [27,31,32]. Malnutrition increases the risk of TB, and conversely, TB can exacerbate the condition of malnutrition. The condition of malnutrition affects the immune function, which predisposes children to the development of TB, and consequently, the illness and inflammatory response worsen malnutrition [31]. In this study, we found that the intervention group had a better nutritional status outcome after completing the TB treatment. These results complement the results of previous studies that supplementation for a short duration of eight weeks did not affect weight gain and improvement of other clinical conditions, but increasing the duration until the end of TB treatment had a positive effect on resolution of fever and cough, as well as improving nutritional status [20].

After completing the TB treatment, none of the study subjects achieved normal vitamin D levels in either group. On the contrary, vitamin D levels in both groups decreased from the category of vitamin D insufficiency to vitamin D deficiency. We suggest that there might be a role of Isoniazid and rifampin as TB drugs that can lead to a higher incidence of hepatotoxicity, affecting the activity and expression of the CYP450 enzyme, thus reduce vitamin D receptor expression secondary to liver damage caused by TB drug [33]. Other studies also proved that the group with decreased vitamin D levels after six months of TB drug showed no change in liver function values, while the group with increased vitamin D levels showed significant improvement in liver function after TB drug for six months [15].

Factors affecting vitamin D levels include genetics, geographical location, tropical climate, skin color, race, food intake, and exposure to sunlight [7,16]. In this study, risk factors such as geographical location, tropical climate, skin color, and race in both study groups did not modify the results because both groups had the same risk factors. Both groups were lived in the West Borneo area, so that the geographical location and climate of the residence were relatively the same. The primary assumption related to skin exposure to sunlight is that all participants were children living in the same area and unfeasible tools to measure all the variability of clothes and daily activities. The exposure to sunlight itself was not measured because there was no standardized method in measuring the amount of sun exposure [34]. This study did not trace the source of nutritional intake, including milk consumed by the subjects, due to the high variability of foods that can be randomly consumed. Although this study did not stratify the socio-economic status of the subjects, most of the subjects’ parents are low educated (lower than the junior high school level), and their occupation was not working or informal workers. Therefore, the type and amount of milk consumed can vary greatly depending on the unstable economic conditions of the parents, assistance or support from donors, and government involvement at certain times.

## CONCLUSIONS

This study adds evidence to the role of vitamin D in improving fever and cough resolution, and improving nutritional status in children with pulmonary TB and vitamin D insufficiency. Determination of adequate supplementation levels of more than 1000 IU requires further research to achieve normal vitamin D levels during the duration of treatment for pulmonary TB in children.
